# Nothing is as practical as a good theory

**DOI:** 10.1007/s40037-012-0022-3

**Published:** 2012-08-08

**Authors:** A. Debbie C. Jaarsma

**Affiliations:** Amsterdam Medical Centre, University of Amsterdam (AMC-UvA), Evidence-Based Education, P.O. Box 22660, 1100 DD Amsterdam, the Netherlands

This is the third issue of *Perspectives on Medical Education*. PME aims to share with you, our reader, the growing body of knowledge and understanding in the broad domain of health professions education. We aim to inspire you to conduct (more) research yourself and to contribute to the building of a coherent body of evidence. Additionally we hope to increase a further understanding of learning in this domain and to better inform the decision making on educational matters in your institute.

To accomplish this PME strives to publish papers that are of high quality, addressing innovative topics and challenging research questions through well-designed studies.

PME values the richness of the health professions educational domain, literally intertwining health professions and education, with authors and readers from a wide variety of backgrounds.

Practitioners and scientists from both the biomedical and the social science domains collaborate in creating the best possible education and to provide the evidence base that informs practice [1]. As the professionals who are involved in medical education come from such diverse backgrounds, PME will cherish and publish papers with a wide variety of perspectives on learning, teaching and assessment, preferably well grounded in theoretical/conceptual frameworks. Perspectives from disciplines such as psychology, education, sociology and anthropology are used more and more to understand and explain learning and to guide the design of educational activities [1].

The diversity displayed by the papers in this issue truly lives up to our aim of sharing that broad range of perspectives that exists in our domain. The papers live up to the title of our journal!

The paper by Van Den Broek et al. on students’ instructions before conducting a Script Concordance Test is of great value for researchers, teachers and others involved in assessments with SCTs. From a theoretical point of view their study helps to enhance our understanding of the SCT and opens up the discussion on test reliability and the influence of validity. From a more practical point of view the study is of interest since it shows how something relatively ‘simple’ as an instruction in how to approach a test, may have large consequences for the validity of that test. The authors have their backgrounds in medicine and psychology, uniquely combining their expertise [2].

Berghmans et al. also inform both theory and practice by bringing back to life the old discussion about directive (e.g., teacher-centred) and facilitative (e.g., student-centred) training approaches and the influence on learning outcomes. In this paper a quasi-experimental design is presented with medical students trained as peer teachers in clinical skills. The trained students were less positive about the facilitative training approach than about the directive approach [3]. Understanding why this is the case would call for more in-depth studies. Observational studies that take into account the purpose of the clinical training and what students actually do could inspire such research.

The discussion ends with the intriguing notion that students’ preferences and expectations should not always be met [3].

A practical study is presented by Boudreau et al. on a faculty development workshop on narrative-based reflective writing. The authors may be commended on their own self-reflectiveness on the outcomes and generalisability of their workshop template [4]. This study inspired me to start using the concept of narrative approaches and elements of the workshop in my own institution.

Henning et al. [5] focused on the well-being of international and domestic students and the potential influence on academic performances, an often neglected topic in the research literature [6]. This is a descriptive study that informs all of us responsible for student affairs to make sure that students (and especially international students) are provided with social networks and that support systems are in place.

The importance of emotional support to students, among other variables that influence students’ motivations, is extensively studied by Rashmi Kusurkar. A short summary of her PhD thesis on Motivation in Medical Students is presented in this issue [7]. Her work sheds a new perspective on medical education valuing student motivation as an important component of curriculum design. Kusurkar’s thesis consists of empirical studies and application to practice studies. A marvellous example of how deepening theoretical understanding intelligently informs practice. A must read for everyone in our domain.

I wish you much joy and academic inspiration in reading this issue. Hopefully it broadens your *perspective*.
